# The advantages of lexicon-based sentiment analysis in an age of machine learning

**DOI:** 10.1371/journal.pone.0313092

**Published:** 2025-01-10

**Authors:** A. Maurits van der Veen, Erik Bleich

**Affiliations:** 1 Department of Government, William & Mary, Williamsburg, Virginia, United States of America; 2 Department of Political Science, Middlebury College, Middlebury, Vermont, United States of America; Educational Testing Service: ETS, UNITED STATES OF AMERICA

## Abstract

Assessing whether texts are positive or negative—sentiment analysis—has wide-ranging applications across many disciplines. Automated approaches make it possible to code near unlimited quantities of texts rapidly, replicably, and with high accuracy. Compared to machine learning and large language model (LLM) approaches, lexicon-based methods may sacrifice some in performance, but in exchange they provide generalizability and domain independence, while crucially offering the possibility of identifying gradations in sentiment. We demonstrate the strong performance of lexica using MultiLexScaled, an approach which averages valences across a number of widely-used general-purpose lexica. We validate it against benchmark datasets from a range of different domains, comparing performance against machine learning and LLM alternatives. In addition, we illustrate the value of identifying fine-grained sentiment levels by showing, in an analysis of pre- and post-9/11 British press coverage of Muslims, that binarized valence metrics give rise to different (and erroneous) conclusions about the nature of the post-9/11 shock as well as about differences between broadsheet and tabloid coverage. The code to apply MultiLexScaled is available online.

## Introduction

The study of sentiment in texts—specifically, whether (and to what degree) a text is positive or negative—has wide-ranging applications. Scholars across the social sciences have long been interested in how best to measure sentiment [e.g. 1, 2], and recent years have seen an explosion of interest, as automated methods have become increasingly accessible and accurate [3, 4]. Many of these methods, however, share three important characteristics that limit their usefulness for general social science applications.

First, innovations in sentiment analysis—including recent breakthroughs using large language models (LLMs)—have often been narrowly application- and domain-specific, which makes them unsuitable for comparisons across applications [5, 6]. For social scientific purposes, maximizing generalizability and comparability across domains has distinct advantages over domain-specific approaches. Second, most methods pay little or no attention to identifying a baseline or reference point. If a text is positive, what does that mean? Positive compared to what? Third, many methods are unable to identify sentiment strength: is a text very positive or just modestly so, and are positive texts more positive than negative texts are negative? As we show in this paper, such reduction of sentiment to a binary classification–positive or negative only–risks drawing the wrong conclusions about patterns and trends in sentiment.

Automated sentiment analysis methods can be classified broadly into three types of approaches: lexicon-based, LLM-based, and machine learning. While the latter may display strong performance on specific datasets, most embody the aforementioned shortcomings that limit generalizability and comparability across applications. This is primarily because they are 1) generally developed or fine-tuned for a single application, and 2) assign texts to one of two possible valences (positive or negative), with little attention paid to what neutrality might mean and none to the degree of positivity or negativity. LLMs do not have these same limitations, but introduce other challenges, among others in terms of built-in biases and challenges in fine tuning, as we discuss below.

There is no doubt that properly designed and trained machine learning models or LLMs can produce better classification performance *within* a specific, well-defined domain than can lexicon-based approaches. However, the successful development and implementation of a high-quality machine learning or LLM classifier demands far more computational resources, as well as human input (in the form of labeled examples).

In contrast, applying pre-existing lexica is very low-cost and can easily be scaled to quickly label hundreds of thousands of texts. The lexicon-based method presented here, MultiLexScaled, does not require any knowledge about the contents of the texts to be coded—and about what might make a particular subset of those texts representative and thus suitable as a training set— nor any prior training on coded texts. Instead, it can be applied directly. To maximize cross-domain generalizability, MultiLexScaled averages scores across eight widely used sentiment dictionaries. These scores are then calibrated, or benchmarked, against a representative corpus of newspaper articles, whose mean sentiment is taken to represent neutrality (neither positive nor negative). Such calibration—which, again, does not require additional human coding— makes it possible to compare degrees of positivity or negativity both within and across corpora.

The remainder of this article is divided into four sections. We begin with a discussion of common approaches to sentiment analysis across the social sciences. The second section outlines the construction of the lexicon-based approach, while the third provides comparative information about its performance against benchmark datasets. Here, we obtain results that match or exceed those of a range of comparable approaches, including machine learning and LLMs. In the fourth section, finally, we demonstrate the specific value of fine-grained sentiment scoring by applying the method to the coverage of Muslims in the British press. Analyzing a corpus of more than 300,000 articles, we demonstrate that relying on binary sentiment indicators would result in drawing the wrong conclusions about the negative shock to sentiment after 9/11, and about divergence in sentiment intensity between broadsheets and the tabloid press. All the lexica and code needed to apply MultiLexScaled are available online; interested users can readily apply the method to their own data and obtain high-quality, domain-independent, fine-grained sentiment data.

## Measuring the valence of texts

Despite work on automated sentiment analysis dating back to the 1960s, many scholars until recently preferred manual coding approaches, which offer greater flexibility and allow a more nuanced set of codes [7]. After all, people remain better at interpreting language than computers; they require no special training to understand grammar, metaphor, sarcasm, etc. [8, 9]. On the other hand, human beings *do* require training in particular coding procedures and rules. Moreover, an individual’s coding practices may change over the course of a coding project, and coders may become tired and introduce inadvertent errors. Nevertheless, for small bodies of text, trained human coders remain the gold standard [10].

Unfortunately, individual coders cannot process large bodies of texts, especially when those texts are longer than just a few lines. Yet relying on multiple coders introduces issues of inter-coder reliability [11–13]. Meanwhile, research suggests that automated approaches can perform just as well as, or even better than, human coders, especially for large corpora. They make up for weaknesses in language understanding by being systematic, adjustable, completely replicable, and not subject to inter-coder reliability concerns [14–16]. In addition, automated coding need not be subject to volume constraints, making sampling unnecessary. However, training or fine-tuning machine learning or LLM models, as well as applying them to a large number of texts, may require extensive computational resources, and thus introduces a different type of volume constraint.

The literature on automated sentiment analysis ranges across disciplines, from the computational to the social sciences [17–21]. Broadly speaking, there are three main strategies: lexicon-based (unsupervised), machine learning (supervised) and LLMs, which can be supervised or unsupervised. In supervised learning approaches, a classifier is trained on a corpus of pre-classified texts, following which it is tasked with classifying similar, unclassified texts. In contrast, unsupervised methods use lexica—lists of words, phrases, etc.—to classify texts, or they rely on knowledge implicitly encoded in an LLM. Social scientists have used both supervised [22–24] and unsupervised [3, 25–27] approaches. In addition, some scholars have applied tools from machine learning to improve lexicon quality [e.g. 28, 29], combining some of the strengths of both approaches.

Supervised approaches are appealing for domain-specific applications, where they can learn localized word usage patterns and sentence construction. However, performance degrades rapidly when they are applied to other domains [5, 30, 31]. More importantly, obtaining optimal performance from supervised learning approaches is not straightforward. First, it is necessary to obtain a representative sample of texts to train on. Depending on the amount of pre-existing domain knowledge, identifying a representative sample may be quite difficult, while learning from a non-representative sample can lead to poor performance [32]. Second, this sample of texts needs to be coded correctly by one or more human coders, which means facing all the challenges associated with human coding [33].

Similar problems also apply to the fine-tuning of LLMs. On the one hand, such models often require fewer labeled samples; on the other they present new challenges such as “catastrophic forgetting”, in which fine-tuning a general model for the sentiment analysis task risks losing many of the strengths of the pre-trained model [34, 35]. In addition, there are often unexpected (and unnoticed) pitfalls in trying to keep the training, validation, and application steps of the machine learning model separate, which may invalidate findings [36]. Nevertheless, a carefully applied machine learning model trained on a correctly-coded representative sample of texts will frequently outperform lexicon-based approaches.

The use of LLMs raises an additional problem even in the absence of domain-specific fine-tuning: that of built-in bias. It is well-established that LLMs will encode the biases of the texts they are trained on; as many of the leading LLMs are trained on vast corpora of online texts, they tend to reflect the racial, gender, religious, and other biases that are widespread in such texts [37]. Among others, and specifically relevant to sentiment analysis applications, they may associate negativity with different groups of people [38]. Moreover, recent studies show that even explicitly de-biased LLMs continue to reflect pernicious biases [39]. If we consider, in addition, the ethical and social risks associated with increased reliance on LLMs [40], the appeal of possibly obtaining slightly improved (albeit quite possibly biased) performance lessens considerably.

Unsupervised approaches offer two key advantages compared to supervised methods. First, they tend to be more domain-independent [41]. They can be applied to a new set of texts without any prior coding or training, and without knowing anything about the distribution of words or valences in these texts. In the LLM context, this is known as zero-shot performance. In addition, although they will perform better on some domains and types of texts than others, performance generally degrades less rapidly across domains than is the case for supervised, fine-tuned models.

Second, lexicon-based models make it possible to measure gradations in sentiment, unlike most supervised models, which classify texts into a small number of categories. As a result, the former are more intrinsically suited to types of questions social scientists often ask, for example about trends over time in average sentiment across large quantities of texts, or about whether one group of texts is more negative than another. In theory, machine learning and LLM approaches can be as fine-grained as one might like, simply by adding as many sentiment classes as desired. For instance, several studies have developed machine learning models for the 25-class Stanford Sentiment Treebank [42, 43] and obtained promising results. However, it remains necessary to pre-determine the number of sentiment classes to use. As the number of classes rises, the classification challenge becomes more difficult; meanwhile, it is impossible to know *ex ante* how many classes would be necessary to prevent the erroneous-conclusion problems identified in the applied section of this paper.

Finally, being able to understand how a sentiment analysis method arrives at its assessment is often crucial in social science applications. With a lexicon-based approach, it is trivial to identify the specific words that drive a sentiment assessment. In contrast, with machine learning approaches this becomes much more difficult, as many of the most popular models are black-box, or provide outputs such as feature weights that are almost impossible to interpret for human observers [44]. LLMs present a comparable problem: although it is possible to query LLMs for the “reasoning” behind a sentiment classification, their nature as “stochastic parrots” means that such reasoning cannot be relied upon [45].

## Method: A lexicon-based, calibrated approach

The potential strength of lexicon-based models is not always realized in practice. A number of widely used general-purpose lexica show less overlap than one would expect: pairwise correlations on positive/negative judgments rarely rise above 0.7 and are frequently much lower [27, 41, 46]. As Chan et al. [47] note, this calls into question just how domain-independent such lexica are, and underscores the importance of validating any method one decides to use. Moreover, their valence scores may be difficult to interpret, and not necessarily symmetric around 0: a value of +1 may not be as positive as a value of -1 is negative [48]. The MultiLexScaled method presented here specifically targets these shortcomings: rather than attempt to improve the performance of a single lexicon on a single, pre-determined corpus, it draws on a set of independently-produced lexica to produce a high-quality measure that maximizes cross-domain validity and replicability, as well as the interpretability of computed valence.

It is worth emphasizing that there is no such thing as an objectively neutral text. Words that may strike one reader (or listener) as purely factual and descriptive might impress someone else as highly value-laden. For this reason, even well-trained human coders working with detailed coding instructions will often disagree on the valence of a text; similarly, an automated sentiment classifier may differ from an individual human’s judgment without necessarily being wrong [33]. Our benchmarking goal, then, is not to identify a specific point that every human reader would recognize as neutral, but rather one that readers, on average, would consider neutral.

To do so, we obtain a representative sample of more than 100,000 articles from leading newspapers in the United States and the United Kingdom, published from 1996 to 2015. We calculate each article’s valence using eight widely used sentiment analysis lexica, each containing lists of positive and negative words. Each measure is calculated based on the presence of words included in that lexicon. In addition, we take into account the presence of modifiers or intensifiers, such as “not” or “very”. Specifically, we use adjust the weight of a valence word if it is immediately preceded by one or more modifiers, following the approach of [49]. The S1 File provides information on the specific adjustments implemented.

Next, we scale the sentiment score by the total number of words in the text, to address the fact that sentiment strength will be diluted by the presence of many non-valence words. Finally, we standardize valences so that the mean for the representative corpus is 0 and the standard deviation 1. As the Central Limit Theorem predicts, the overall distribution of valences in the representative corpus approximates a normal distribution for each individual lexicon. Finally, the eight resulting measures are averaged, and rescaled once more to a standard deviation of 1. The resulting valence scores are easily and intuitively interpretable in standard deviation units.

The same steps of calculating, standardization, averaging, and scaling can then be applied to any other text, using the standardization parameters derived from the representative corpus. This produces a valence that can be interpreted relative to the benchmark. Note that this approach can be used to develop any number of different benchmarks: for example, some might prefer a single-country benchmark for their purposes; others might prefer to benchmark against a particular type of texts, such as speeches or social media posts. Our goal here is to develop a benchmark that is relevant beyond a single national context, increasing its general applicability. As we shall see below, the differences between the US and the British parts of the representative corpora are minor.

The eight lexica used in MultiLexScaled have been deliberately selected to have a wide range of different characteristics, in order to maximize their diversity without sacrificing generalizability. The two that have been in use longest were produced in the early 2000s by computer scientists developing new tools for automated sentiment analysis [50, 51]. They were constructed based on earlier work by computer scientists. Interestingly, each contains roughly twice as many negative as positive words. One simply contains lists of positive and negative words (all positive/negative words are equally positive/negative); the other ranks sentiment intensity from +/-0.175 to +/-1.

The other six lexica are newer, and provide even more diversity. Two rank sentiment intensity; the other four simply identify words as positive or negative. Two of them assign valence levels to words by relying on human coders recruited through Amazon’s Mechanical Turk [52, 53]. One automatically propagates valences through a semantic and syntactic network, WordNet, starting with paradigmatically positive and negative words (“good”, “bad”, etc.) [54]. For the other three, the constructors of the lexicon assigned valences themselves. Two of them include not just words but also word stems, or roots: “abhor*” will capture “abhor”, but also “abhors”, “abhorred”, “abhorrent”, etc. While most lexica were developed by computer scientists and computational linguists, one was produced by political scientists [27], and one by a commercial company selling text analysis software [55]. All contain more negative than positive terms, and they range in size from under 4,000 to over 24,000 total terms included.

Details about each of the lexica are provided in the S1 File. Here we briefly describe one of the lexica commonly used by social scientists, LexicoderSD, to give a general sense of the contents of these lexica. LexicoderSD was developed to code political news texts [27]. It contains all the words included in two earlier sentiment dictionaries, the General Inquirer [56] and the Regressive Imagery Dictionary [57] along with Roget’s Thesaurus as long as those sources agree on the valence. It includes wildcards, accepting any word endings for a given word stem. In total, it contains 1608 positive words, of which 1040 are word stems, and 2745 negative words, of which 1958 are word stems.

## Validation

### Representativeness

The benchmarking corpus used to develop the scaling parameters for MultiLexScaled is intended to be broadly representative of anglophone print media; in particular, the British and American newspaper markets. Although classification performance (assessed below) is more important overall, it is nonetheless necessary to verify that the neutral point in the representative corpus is not too far off from the mean valence of articles in any particular newspaper. If individual newspaper sources were to diverge considerably, this would call into question our ability to draw conclusions about how a particular valence level compares to the print media in general. We do not expect the mean valence for every media source to be exactly 0—individual newspapers are bound to diverge somewhat from a multi-source, cross-national average—but a mean sentiment far from the benchmark neutrality would be cause for concern.

We calculate the valence for all articles published in four different newspapers in the corpus, selected to cover a range of different types of outlets: a left-leaning (*Observer*) and a right-leaning (*Sunday Times*) paper from the UK, as well as a tabloid paper from that country (*Daily/Sunday Mail*), plus a general-audience title from the US (*USA Today*). For each of these, we consider four different three-year periods: two during the 1996–2015 period constituting the representative corpus, plus one just before and one right after. This allows us to identify any over-time trends present and to verify that the 1996–2015 period is not an outlier in some way.

Table 1 shows the results. Average valences for the 3-year periods range from -0.26 to +0.20, or at most a quarter of a standard deviation away from the overall representative corpus mean. The most consistent “outlier” from the representative corpus mean is the tabloid paper, which remains at -0.17 or lower throughout. The other papers all trend slightly more positive over time, but the greatest shift over the quarter century covered remains minor, again at just about a quarter of a standard deviation (for the *Sunday Times*). These figures indicate that the representative corpus average does not hide wide variation across different titles, and that over-time trends are comparatively minor. In short: the article valences in the representative corpus can indeed be considered representative of the valences in a wide variety of types of newspapers across location and time, even for periods prior to and after the years for which it was collected.

**Table 1:** Mean article valences by paper, 3-year averages.

Paper	1993–1995	2001–2003	2008–2010	2016–2018
*Daily & Sunday Mail*	-0.24 (113,705)	-0.22 (159,984)	-0.17 (202,058)	-0.26 (126,349)
*Observer*	-0.13 (37,814)	-0.07 (54,634)	-0.08 (86,303)	0.02 (21,507)
*Sunday Times*	-0.07 (47,609)	0.02 (75,459)	0.05 (139,445)	0.20 (130,349)
*USA Today*	-0.08 (93,365)	-0.05 (65,672)	0.05 (53,933)	0.07 (38,061)

Number of articles in parentheses.

To verify that our benchmarking is relevant beyond the United States and the United Kingdom, we generate analogous representative corpora from five or six widely read newspapers in each of three additional Anglophone countries: Australia, Canada, and New Zealand. Table 2 displays the results for all five national representative corpora. As the table makes clear, the differences between the US, UK, and Canadian national corpora are negligible: we could substitute any of these single-nation representative corpora for the two-country US-UK benchmark, with only very minor impact. For Australia and New Zealand, the mean valence is more positive, but even in these countries the deviation from the UK-US neutral point is less than one seventh of a standard deviation.

**Table 2:** Valence of national representative corpora.

	N	Mean valence
United Kingdom	59,404	-0.014
United States	48,283	0.018
Canada	22,860	0.014
Australia	24,114	0.132
New Zealand	7,455	0.136

Measured using the two-country (US & UK) benchmark.

### Performance

Even if the calibration reflects the mean sentiment of Anglophone print media in a range of countries, this still does not tell us whether that same sentiment would in fact be interpreted as neutral by the average reader. It may be the case, for instance, that news reporting is systematically negative [58]. To assess whether the calibration’s zero point accurately indicates neutrality, we test our ability to classify texts with known sentiment polarity, drawn from several different domains.

Scholars have constructed numerous test corpora, the most widely used of which are constructed from online reviews of products and movies [30, 59]. Such corpora are appealing because the authors of such reviews often assign a grade or a ranking, obviating the need for additional human coding. In addition, as online reviews proliferate, these test corpora can be quite large, making it less likely that a model’s performance will be driven by idiosyncratic features of a small number of texts. However, these test corpora also pose a hard test for our method, which was neither designed nor benchmarked to classify product or entertainment descriptions.

One widely used test corpus contains 50,000 reviews from the Internet Movie Database (imdb), each labeled as either positive or negative (25,000 of each category), based on the movie ratings assigned by the commenters themselves [60]. For each of these reviews, we calculate valences using MultiLexScaled. Since the positive and negative categories are balanced in the dataset, we simply report the percentage classified correctly. The top row in Table 3 shows that the method gets over 75% of the valence polarities of the individual reviews correct. In addition, further analysis indicates that mis-classifications are evenly distributed between positive and negative reviews, and disproportionately occur for reviews that are just weakly positive or negative (i.e. closer to neutral).

**Table 3:** Validation test results on the imdb corpus.

Adjustments to neutral point	% correct	Worst lexicon	% correct	Best lexicon	% correct
-	75.68	labMT	67.97	SO-CAL	78.13
Internal calibration	75.67	labMT	69.55	SO-CAL	78.18
Optimal calibration	75.71	labMT	69.59	SO-CAL	78.25

Speaking directly to the question of calibration, the two bottom rows show two alternative methods of determining neutrality: using the imdb corpus’ own mean valence as the neutral point, and selecting the neutral point so as to maximize the percentage correctly classified as positive or negative. The differences are marginal, and for the imdb mean the performance actually deteriorates very slightly. In other words: the MultiLexScaled US-UK neutral point, derived from a completely different dataset (not just containing different texts, but texts primarily about subjects having nothing to do with movie reviews) allows us to achieve a classification performance that is effectively identical to the best we could achieve by tailoring to this particular corpus. This result supports our argument that the MultiLexScaled method is domain-independent. In addition, it indicates that newspaper articles do not, in fact, have a negativity bias overall (at least, not compared to user-written movie reviews).

Table 3 also provides information about the worst- and best-performing lexica for this corpus, among the eight lexica used in the method. While averaging performs slightly less well than using the SO-CAL lexicon by itself (that lexicon was specifically designed to analyze reviews), it outperforms all seven other lexica; moreover, the performance loss compared to the top individual lexicon is minor. The performance range across the lexica underscores the importance of averaging their results: the loss from averaging across multiple lexica (about 2.5% compared to the best performer) is rather smaller than the improvement on the worst performer (about 6%). Averaging becomes particularly important when we do not know *ex ante* which lexicon might perform best, as is usually the case with social science applications.

Since this is a widely used test corpus, we can compare the method’s performance against other automated classifiers in the literature. Supervised machine learning classifiers specifically trained on this imdb corpus can reach accuracy levels slightly over 90% [61]. Meanwhile, Rice and Zorn show that a domain-specific lexicon generated by identifying the most unambiguously positive and negative words in the imdb dataset correctly identifies polarity 80% of the time [28]. For those interested only in identifying the valence of individual texts within a single, specific domain and with the expertise to set up and train a machine learning model, a 15 percentage point performance increase likely justifies the additional investment of time and resources that would require. However, similar performance gains are far from guaranteed, as we show below.

More importantly, social scientists are rarely interested in identifying the valence of individual texts; instead, the focus is generally on groups of texts and on broader patterns or trends [22]. To give just a few recent examples, Rhodes and Vayo track the degree of positivity and negativity in hundreds of presidential campaign speeches in the United States over time [62]; Paxton, Velasco and Ressler analyze the mission statements of thousands of non-profits to see whether the positivity or negativity of those statements is associated with different levels of donations and volunteering [3]; and Shapiro, Sudhof and Wilson track trends in the sentiment of economic news across several hundred thousand newspaper articles [4]. For such applications, what matters most is that *overall* patterns are measured accurately.

This means that a better test of sentiment analysis methods for the social sciences is to assess their classification performance on groups of texts: they should not be systematically biased in one direction (positive or negative) or another, and, relatedly, should be highly accurate for comparatively small subsets of texts, such as would result from a division of a full corpus of texts into smaller sets based on other factors of interest (in the preceding examples: presidential candidates, types of non-profits, or dates, for example). To test MultiLexScaled accordingly, we run simulations in which we randomly select N movie reviews from the imdb dataset with the same polarity (positive or negative), average their calculated valence, and compare this to the “true” polarity. In order to eliminate the possibility that the method is biased in one direction, we separate the two categories. For each simulation, we select 1,000 sets of reviews (each set composed of N reviews randomly drawn without replacement), and we repeat each simulation 1,000 times in order to obtain confidence intervals. Table 4 displays the results.

**Table 4:** Bootstrap simulation performance, sets of N reviews.

N	Negative accuracy	95% lower	upper	Positive accuracy	95% lower	upper	Overall Accuracy	95% lower	upper
3	0.89	0.87	0.91	0.87	0.85	0.89	0.88	0.85	0.91
6	0.96	0.95	0.97	0.94	0.93	0.96	0.95	0.93	0.97
12	0.99	0.99	1.00	0.99	0.98	0.99	0.99	0.98	1.00
25	1.00	1.00	1.00	1.00	1.00	1.00	1.00	1.00	1.00

1000 draws of 1000 observations, showing the derived 95% confidence intervals.

As the table shows, the approach performs very well on even small sets of texts. For 6 texts of the same polarity, the entirety of the confidence interval is already over 90% accuracy. Accuracy continues to rise rapidly as N grows, and by 12 texts, performance is at 99%. In sum, for even modest-sized sets of articles, MultiLexScaled identifies polarity with an accuracy that matches or exceeds that of the best machine learning classifiers specifically trained on this corpus.

We use the same approach to test the method’s ability to identify gradations of positivity and negativity. As outlined earlier, the ability to do so is very important in the social sciences, yet sentiment analysis work to date has focused primarily on polarity alone. The imdb corpus includes additional information about the rating a user assigned to the movie they reviewed. It includes rating values of 1–4 (from very to mildly negative) and 7–10 (from mildly to very positive). Table 5 shows the average valence MultiLexScaled assigns to each rating level: a 1-point increase in rating is associated with a valence increase of about 0.15–0.20, and t-tests indicate that the difference between adjacent mean valence levels is statistically significant. In other words, MultiLexScaled is able to reliably distinguish even quite small differences in average tone *within* a particular polarity (positive as well as negative). Strikingly, this holds even though the reviews are scored by hundreds of different reviewers, with no standardized coding criteria.

**Table 5:** Average valence by imdb movie review rating.

Rating	N	Mean valence	Difference test	t-stat	p-value
1	10,122	-1.11			
2	4,586	-0.88	1 & 2	-11.87	< 0.00001
3	4,961	-0.71	2 & 3	-7.81	< 0.00001
4	5,331	-0.55	3 & 4	-7.91	< 0.00001
7	4,803	0.27	7 & 8	-8.61	< 0.00001
8	5,859	0.47	8 & 9	-4.67	< 0.00001
9	4,607	0.58	9 & 10	-6.35	< 0.00001
10	9,731	0.73			

Final 3 columns show t-test results for difference between adjacent rating levels.

The S1 File provides additional validation tests on several corpora used in the SentiBench benchmarking collection [63]. Across all of these validation tests, both the best- and the worst-performing lexica within MultiLexScaled vary, underscoring the value of averaging across multiple lexica for obtaining domain independence.

We finish the validation section by testing MultiLexScaled on two test corpora used in recent studies that compare the strengths of different classification methods. First, Barberá et al. [64] use a corpus of 420 segments of newspaper articles about the economy to compare the performance of a machine learning classifier to that of several different lexica. An error in their machine learning set-up leads to an overly positive assessment of the machine learning classifier [65]. After correcting for this error, their classifier correctly labels 68.1% of the texts, while the accuracy of the best-performing lexicon they test—Lexicoder, which is also one of the lexica included in MultiLexScaled—is just 61.2%. However, MultiLexScaled correctly classifies 65.7% of the texts, just 2.4% below that of the machine learning classifier. Moreover, given the small size of the test set, these performance figures depend heavily on the specific articles included. Bootstrapping by sampling with replacement 420 texts from the original test set permits the creation of confidence intervals around these values. For the machine learning classifier, the 95% range is 63.6%-72.4%; for MultiLexScaled it is 61.2%-70.2%. In other words, the overlap is more than two thirds of the range. Indeed, in about 15% of the bootstrap samples, MultiLexScaled outperforms the machine learning classifier, demonstrating that even a well-trained machine learning classifier can be outperformed by a dictionary-based method. Additional details about the analysis appear in the S1 File.

Finally, we classify a set of 1500 short text snippets from the website StackOverflow [66]. These were used in a recent study assessing how state-of-the-art large language models perform at zero-shot (i.e. no additional fine-tuning) or few-shot (using a few coded examples) sentiment classification [67], which makes it possible for us to compare the performance of the lexicon-based approach to those LLMs. The study tested several different prompts on three different open-source LLMs, all based on the Llama 2 model [68]: Llama 2-Chat, Vicuna [69] and WizardLM [70]. The StackOverflow dataset contains 131 negative sentences, 178 positive sentences, and 1,191 neutral sentences.

Since the MultiLexScaled approach is designed for longer texts, where the sentiment impact of individual words is attenuated, when we apply it to short sentences, it produces larger sentiment scores. Accordingly, we set symmetric bounds around 0 of -2 and +2 standard deviation units for the ‘neutral’ category. With an unbalanced dataset, the proportion of sentences labeled correctly is not a helpful metric. Instead, we look at F1 scores, which take into account precision and recall for each category, both micro-F1 (the average across the individual F1 scores for each possible category), and macro-F1 (the overall F1 score). For MultiLexScaled, these values are 0.69 and 0.53, respectively. For zero-shot LLMs classification, the analogous F1 values range from 0.61–0.82 and 0.41–0.59, respectively. For the few-shot classification, the ranges are 0.42–0.83 and 0.42–0.65 [67]. In other words, the lexicon-based performance is squarely in the middle of the LLM performances. Indeed, for some of the LLM prompts used, MultiLexScaled outperforms two of the three LLMs (Llama 2-chat and WizardLM).

The implications of all these validation tests are significant. First, our lexicon-based approach is remarkably robust and domain-independent: the datasets used here range from movie reviews, to snippets of StackOverflow comments, to economic news. Second, for “zero-shot” sentiment analysis, i.e. without domain-specific pre-training, our lexicon-based approach performs comparably to both machine learning approaches and LLM models. Third, the performance of the lexicon-based approach increases rapidly as not individual texts but rather groups of documents are considered—the usual application in the social sciences—reaching levels where it can easily match the performance of ML or LLM models. Finally, as a methodological point, many commonly used datasets are comparatively small, and published differences in performance may be driven by an even smaller subset of the dataset; it is therefore important to look beyond point comparisons of performance and instead get estimates for performance ranges using approaches such as bootstrapping, as well as compare performance across multiple datasets, as we do here.

## Application: Newspaper coverage of Islam and Muslims

When social scientists turn to automated text analysis, it is generally because they want to code large quantities of texts, making fully manual coding infeasible. While well-trained machine learning models can outperform lexicon-based approaches at classifying individual texts, this advantage rapidly disappears for even quite small groups of texts. Moreover, the valence measures lexica produce are comparable across applications, as long as the calibration remains constant. This means we can directly compare the tone of texts across domains, time periods, national contexts, etc., which is invaluable for social science applications.

Of no less importance, lexicon-based approaches make it possible to identify gradations of positivity and negativity, something that is far more difficult to achieve with machine learning models, and is effectively impossible without a lot of additional fine-tuning. The MultiLexScaled approach provides reliable and valid fine-grained results with no *ex ante* information about the particular domain, the nature of the data (long or short texts, formal or online writing, etc.). Being able to identify not only *whether* a text is positive or negative, but also *how* positive or negative, is crucial in many social science applications of sentiment analysis. While it is possible to approximate overall positivity or negativity by averaging across texts, this will often lead to the wrong conclusions, as we demonstrate here.

Sentiment analyses of the media’s coverage of Muslims have consistently found that reporting is negative in tone [e.g. 71–75]. However, they rarely delve further into patterns or comparisons, despite the obvious value of reliable evidence about differences across sources or trends over time in the tone of coverage. More fine-grained information is not only of intrinsic interest; is important, too, because of the causal impact of the media on real-world outcomes regarding minority issues, including attitudes, voting intentions, and political party agendas [76–79].

The analysis here aims to address two key issues raised by the scholarly literature: the impact of a major event such as 9/11, and differences between broadsheets and tabloids. Claims that media coverage became much more negative after September 11, 2001 are common [e.g. 80]. However, others find that headlines and front page stories may have become more positive [81, 82]. Differences in coverage between broadsheets and tabloids are well-established both in general [83] and regarding coverage of Islam in particular [84, 85]. However, there has been little research into the size of these differences or into trends over time. In short, along with the possibly divergent trends in broadsheets and tabloids, the short- and longer-term effects of 9/11 remain incompletely understood.

In order to assess British newspaper coverage of Islam and Muslims over time, we collect all articles containing the word stems ‘Islam’, ‘Muslim’, or ‘Moslem’ anywhere in the headline or in the body of the article. We draw on the same set of newspapers used to construct the British part of our representative corpus, for the period 1996–2016. After removing duplicates and articles erroneously captured, the corpus contains 318,437 articles, with over 215 million words. Table 6 presents an overview of the contribution of different publications to the corpus, along with the valence data for each source’s articles about Muslims and Islam. The tone of coverage is strongly negative, with an average valence of -1.07 across all articles. This means that the average article mentioning Muslims or Islam is, looking back to Table 5, about as negative as the average movie review to which a reviewer assigned the most negative rating.

**Table 6:** Valence of Muslim coverage in British newspapers 1996–2016.

Sources	N	Overall	pre-9/11	post-9/11	change
***Overall***	*318*,*437*	*-1*.*07*	*-0*.*94*	*-1*.*09*	-0.15
***Tabloids***					
*Daily & Sunday Mirror*	16,058	-1.67	-1.27	-1.72	-0.45
*Daily Mail and Mail on Sunday*	19,611	-1.07	-0.84	-1.09	-0.25
*Daily Record & Sunday Mail*	8,184	-1.55	-1.40	-1.58	-0.18
*Daily Star & Daily Star Sunday*	7,181	-1.59	-1.39	-1.59	-0.20
*Evening Standard*	13,883	-1.12	-0.94	-1.14	-0.20
*Express*	11,422	-1.28	-0.86	-1.30	-0.44
*News of the World*	1,430	-1.47	-1.25	-1.49	-0.24
*People*	1,634	-1.54	-0.83	-1.60	-0.77
*Sun*	17,526	-1.60	-1.31	-1.61	-0.31
Average		*-1*.*43*	*-1*.*12*	*-1*.*46*	*-0*.*34*
***Broadsheets***					
*Daily Telegraph & Sunday Telegraph*	32,229	-0.95	-0.91	-0.95	-0.04
*Financial Times*	30,586	-0.84	-0.89	-0.83	0.06
*Guardian*	48,210	-0.87	-1.01	-0.85	0.16
*i-independent print ltd*	6,570	-1.24	n.a.		
*Independent*	39,717	-1.06	-0.96	-1.08	-0.12
*Observer*	9,457	-0.77	-0.83	-0.76	0.07
*Times & Sunday Times*	54,739	-0.92	-0.82	-0.94	-0.12
Average		*-0*.*95*	*-0*.*90*	*-0*.*90*	*0*.*00*

The table also provides the information necessary to shed light on differences between broadsheets and tabloids, as well as on the impact of 9/11. Every tabloid in our corpus is, on average, more negative than all of the broadsheets except for the *Independent*’s sister paper “i.” Indeed, several of the most negative tabloids are more than twice as negative as the least negative broadsheet, the Sunday paper *Observer*. Next, as the first row of the table shows, the period since 9/11 features more negative coverage overall. However, the increased negativity is entirely due to dramatically more negative coverage in tabloids: across the broadsheets that were already being published before 2001, the average trend is effectively flat.

**Fig 1:**
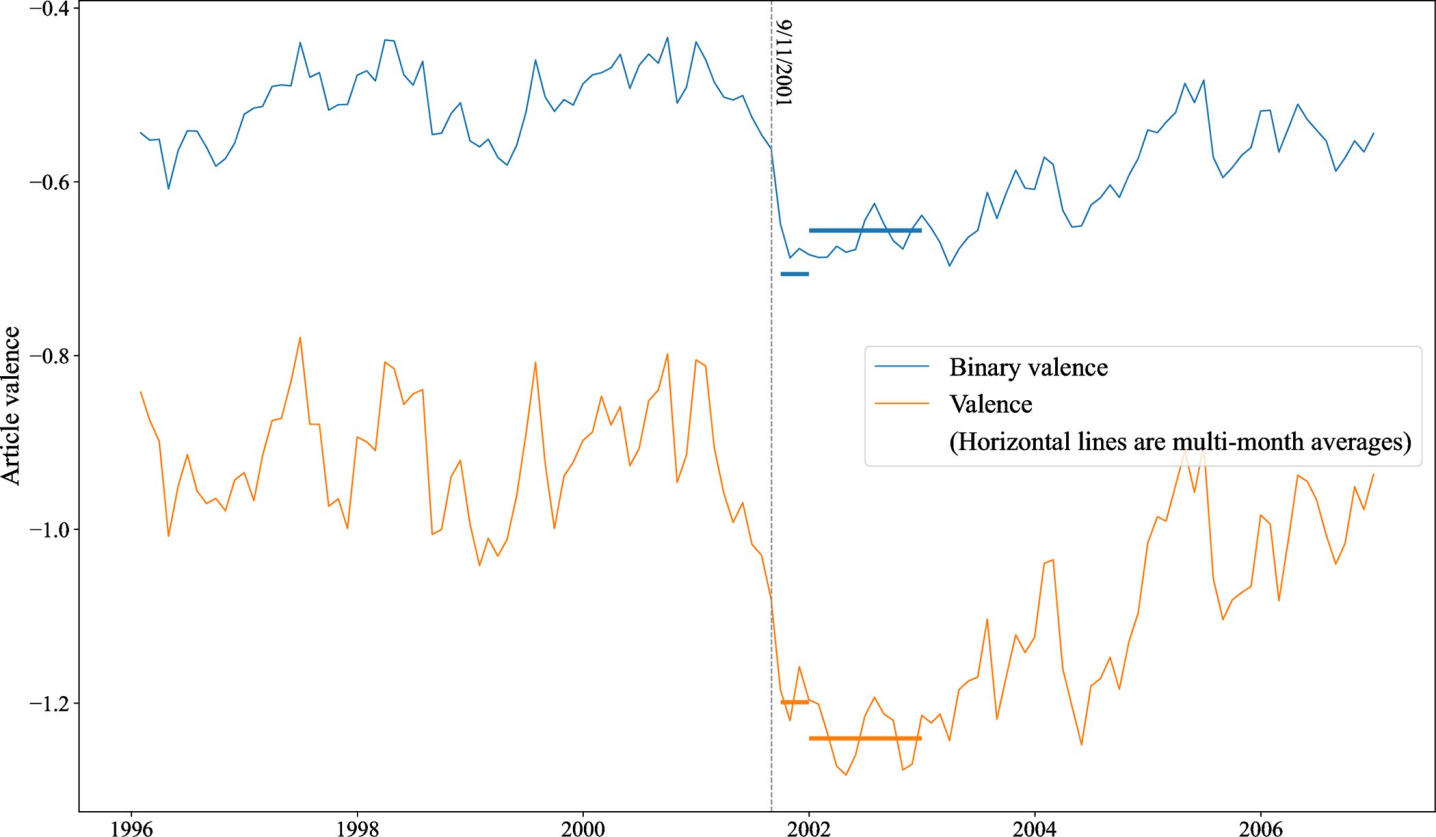
Average monthly valence (smoothed with a 5-month exponential moving average). Note: monthly valence = average of valences of all articles published that month.

These data do not directly speak to the question of whether having fine-grained sentiment data makes a difference. How similar would patterns look if we simply classified articles as positive (+1) or negative (-1) and aggregated those scores across articles published at different points in time? Fig 1 plots the overall trend for the five years before and after 9/11; the upper line represents binarized valence data; the lower line the original MultiLexScaled scores. It is immediately clear that binary positive/negative labeling dramatically understates the negativity of media coverage overall: in reality, negative texts are much more negative than positive ones are positive.

In addition, while the overall ups and downs of the two lines are strongly correlated, they are sufficiently different that analyses can produce different conclusions. Consider, for example, the question of how quickly the tone of media coverage bounced back after 9/11. The two thick horizontal lines crossing each of the over-time trend lines reflect the overall average valence, respectively, for October-December 2001 and all of 2002. Considering only binarized valence would lead one to conclude, erroneously, that coverage bounced back almost immediately, with average valence in 2002 higher than that for the last part of 2001. If we take into account how negative the negative coverage is, in contrast, it becomes clear that coverage in 2002 was actually *more* negative than at the end of 2001.

**Fig 2:**
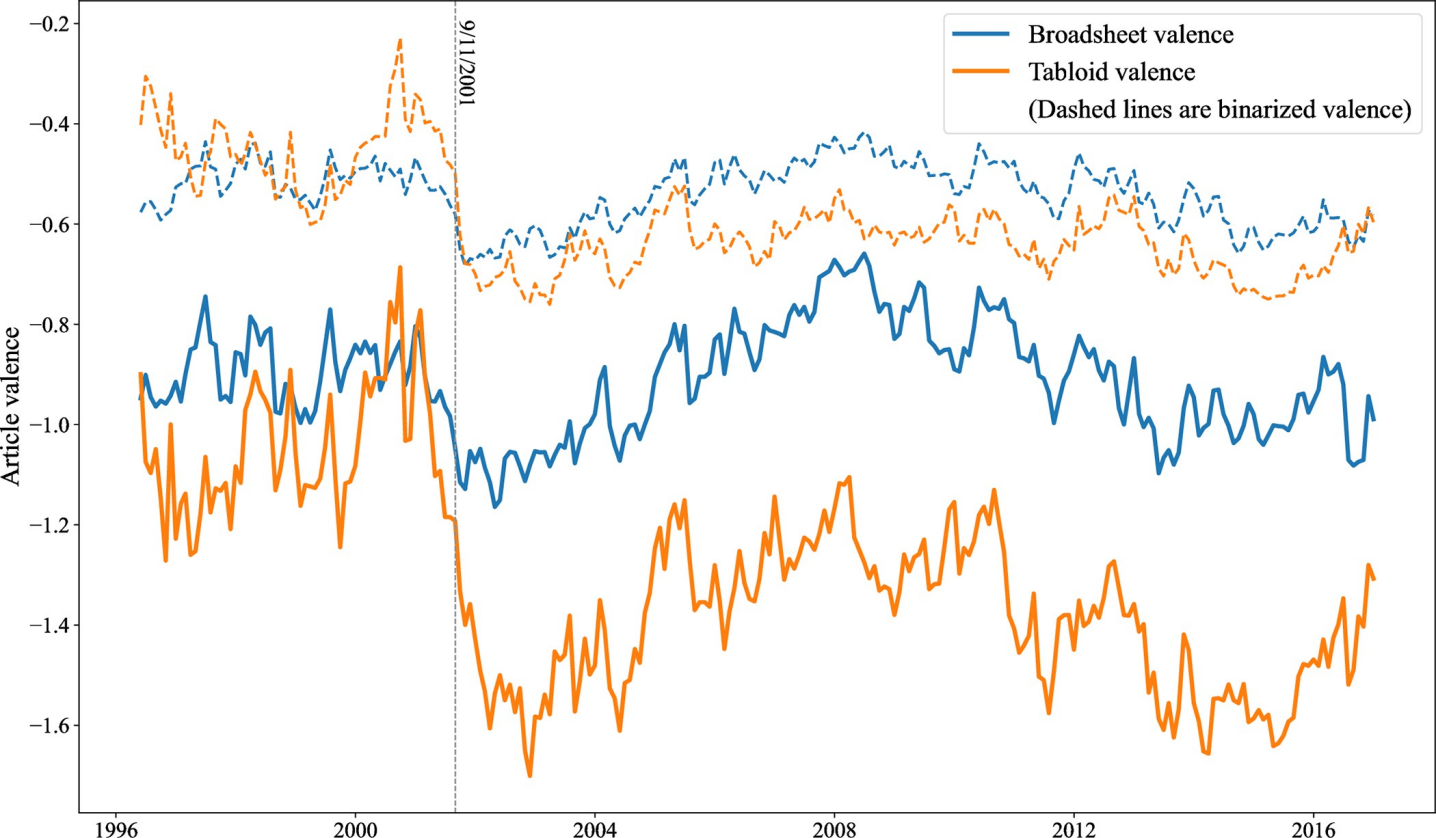
Broadsheet and tabloid monthly valence (5-month exponential moving avg). Note: monthly valence = average of all articles published that month.

Fig 2 shows the same data, but for the full 1996–2016 period, and plotting broadsheets and tabloids separately. The figure underscores the disproportionate impact of 9/11 on the coverage of Muslims in British tabloids. While tabloids already tended to be more negative than broadsheets prior to 9/11, that event really did mark a sea change, introducing a sizeable gap in the average tone of coverage that has persisted going forward. However, if based on binarized valences only, one would again reach the opposite (and erroneous) conclusion. In the dashed, upper lines on the figure, it looks as though tabloids and broadsheets continue to be quite similar in the tone of their coverage after 9/11, with the two lines touching each other repeatedly, and effectively overlapping for the last part of 2016. The binarized valence data completely obscures how much more dramatically negative tabloid coverage of Muslims became after 9/11.

## Conclusion

Sentiment analysis has been of interest to social scientists since well before the term itself gained currency (Pride and Richards 1974, Terry and Mendenhall 1974). Over the past decade, the surge in availability of digitized texts, combined with the increased ability of computers to process large quantities of texts, have spurred new scholarly interest in this approach. Benchmarking, calibration, and comparability of findings have been fundamental challenges that predate automation and digitization: What constitutes a neutral text? Just how positive is a positive text? And what would that level of positivity look like in another domain? The vast majority of sentiment analysis applications, whether they rely on manual or automated coding, are specifically designed to apply only to one particular corpus, limiting the generalizability of findings.

In contrast, the lexicon-based approach presented here, MultiLexScaled, derives calibration parameters from a representative sample of more than 100,000 newspaper articles published in several dozen American and British newspapers. The valences of texts in the representative corpus are standardized to have a mean of 0 and a standard deviation of 1. These calibration parameters can then be applied to any other text, generating a measure of valence expressed in standard deviation units relative to the representative corpus benchmark. Averaging the sentiment values produced by eight different, widely used, lexica helps produce domain independence, while obtaining performance close to that of the best individual lexicon. Moreover, since it is impossible to know *ex ante* the best individual lexicon for a given application, this strategy eliminates the risk of choosing a poorly suited lexicon due to a lack of data to guide lexicon choice [cf. 47]. While the benchmarking corpus consists of newspaper texts, the validation tests showed that MultiLexScaled works equally well on other types of texts, such as movie reviews, or online comments.

We illustrate the value of obtaining fine-grained sentiment measures by applying the method to the coverage of Muslims in the British press, from 1996–2016. The results not only help clarify some open questions in the literature on media coverage of Muslims and other minorities; they also underscore the risks inherent in relying on binarized (positive or negative only) sentiment values. First, press coverage in the immediate aftermath of 9/11, while of course very negative, would become more negative still during 2002. However, this is due to negative articles becoming more negative, not to a shift in the balance between positive and negative articles. Looking at binarized sentiment trends suggests, erroneously, that the coverage in 2002 was actually more positive, on average, than in the immediate aftermath of 9/11. Second, the widely criticized “tabloidization” of the press [e.g. 86] is not reflected in a convergence between broadsheets and tabloids in their coverage of Muslims and Islam, although looking at binarized valences alone might suggest that to be the case. Instead, the tone of broadsheet and tabloid coverage has diverged significantly since 9/11: while that event caused a measurable negative shock in coverage overall, that increase in negativity is due entirely to much more negative coverage in the tabloid press.

## Replication data

Code and calibration parameters necessary to apply MultiLexScaled “off-the-shelf” to a new corpus are available online, at https://github.com/amaurits/MultiLexScaled. Replication code for the analyses is also available at this repository, along with the sentiment calibration data. The search criteria used to obtain the newspaper articles are described in the S1 File; metadata for the resulting representative corpora of newspaper articles from different countries and the corpus of British Muslim coverage, can be found in the Github repository; LexisNexis and Factiva policies prohibit the sharing of the article texts.

## Supporting information

S1 File The supporting information file provides additional details on the newspaper and selection for the representative corpora and the British Muslim corpus; text pre-processing; valence calculation; and further validation tests. (DOCX)

## References

[1] Holsti OR. An Adaptation of the “General Inquirer” for the Systematic Analysis of Political Documents. Behavioral Science; Baltimore, Md. 1964 Oct 1;9(4):382–7. doi: 10.1002/bs.3830090412 5888791

[2] Singer JD. Data-Making in International Relations. Behavioral Science; Baltimore, Md. 1965 Jan 1;10(1):68–80. doi: 10.1002/bs.3830100107 14285223

[3] Paxton P, Velasco K, Ressler RW. Does Use of Emotion Increase Donations and Volunteers for Nonprofits? Am Sociol Rev. 2020 Dec 1;85(6):1051–83. doi: 10.1177/0003122420960104 38737816 PMC11086700

[4] Shapiro AH, Sudhof M, Wilson DJ. Measuring news sentiment. Journal of Econometrics. 2021;24.

[5] Loughran T, Mcdonald B. When Is a Liability Not a Liability? Textual Analysis, Dictionaries, and 10-Ks. The Journal of Finance. 2011 Feb;66(1):35–65.

[6] Deng S, Sinha AP, Zhao H. Adapting sentiment lexicons to domain-specific social media texts. Decision Support Systems. 2017 Feb;94:65–76.

[7] Zamith R, Lewis SC. Content Analysis and the Algorithmic Coder: What Computational Social Science Means for Traditional Modes of Media Analysis. The ANNALS of the American Academy of Political and Social Science. 2015 May 1;659(1):307–18.

[8] Conway M. The Subjective Precision of Computers: A Methodological Comparison with Human Coding in Content Analysis. Journalism & Mass Communication Quarterly. 2006 Mar 1;83(1):186–200.

[9] Sjøvaag H, Stavelin E. Web media and the quantitative content analysis: Methodological challenges in measuring online news content. Convergence. 2012 May 1;18(2):215–29.

[10] van Atteveldt W, van der Velden MACG, Boukes M. The Validity of Sentiment Analysis:Comparing Manual Annotation, Crowd-Coding, Dictionary Approaches, and Machine Learning Algorithms. Communication Methods and Measures. 2021 Jan 28;1–20.

[11] Kalton G, Stowell R. A Study of Coder Variability. Journal of the Royal Statistical Society Series C (Applied Statistics). 1979;28(3):276–89.

[12] Lombard M, Snyder-Duch J, Bracken CC. Content Analysis in Mass Communication: Assessment and Reporting of Intercoder Reliability. Hum Commun Res. 2002 Oct 1;28(4):587–604.

[13] Mikhaylov S, Laver M, Benoit KR. Coder Reliability and Misclassification in the Human Coding of Party Manifestos. Polit anal. 2012;20(1):78–91.

[14] Aust PJ. Communicated values as indicators of organizational identity: A method for organizational assessment and its application in a case study. Communication Studies. 2004 Dec 1;55(4):515–34.

[15] King G, Lowe W. An Automated Information Extraction Tool for International Conflict Data with Performance as Good as Human Coders: A Rare Events Evaluation Design. International Organization. 2003 ed;57(3):617–42.

[16] Rosenberg SD, Schnurr PP, Oxman TE. Content Analysis: A Comparison of Manual and Computerized Systems. Journal of Personality Assessment. 1990 Mar 1;54(1–2):298–310. doi: 10.1080/00223891.1990.9673995 2179519

[17] Benamara F, Taboada M, Mathieu Y. Evaluative Language Beyond Bags of Words: Linguistic Insights and Computational Applications. Computational Linguistics. 2017 Apr;43(1):201–64.

[18] Liu B, Zhang L. A survey of opinion mining and sentiment analysis. In: Aggarwal CC, Zhai C, editors. Mining text data. New York, NY: Springer; 2012. p. 415–63.

[19] Piryani R, Madhavi D, Singh VK. Analytical mapping of opinion mining and sentiment analysis research during 2000–2015. Information Processing & Management. 2017 Jan;53(1):122–50.

[20] Ravi K, Ravi V. A survey on opinion mining and sentiment analysis: Tasks, approaches and applications. Knowledge-Based Systems. 2015 Nov;89:14–46.

[21] Garcia D, Pellert M, Lasser J, Metzler H. Social media emotion macroscopes reflect emotional experiences in society at large. arXiv:210713236 [cs] [Internet]. 2021 Jul 28 [cited 2021 Aug 3]; Available from: http://arxiv.org/abs/2107.13236

[22] Hopkins DJ, King G. A Method of Automated Nonparametric Content Analysis for Social Science. American Journal of Political Science. 2010 Jan;54(1):229–47.

[23] Jamal AA, Keohane RO, Romney D, Tingley D. Anti-Americanism and Anti-Interventionism in Arabic Twitter Discourses. Perspect polit. 2015 Mar;13(1):55–73.

[24] van Atteveldt W, Kleinnijenhuis J, Ruigrok N, Schlobach S. Good News or Bad News? Conducting Sentiment Analysis on Dutch Text to Distinguish Between Positive and Negative Relations. Journal of Information Technology & Politics. 2008 Jul 14;5(1):73–94.

[25] Burscher B, Vliegenthart R, de Vreese CH. Frames Beyond Words: Applying Cluster and Sentiment Analysis to News Coverage of the Nuclear Power Issue. Social Science Computer Review. 2016 Oct;34(5):530–45.

[26] Soroka SN, Stecula DA, Wlezien C. It’s (Change in) the (Future) Economy, Stupid: Economic Indicators, the Media, and Public Opinion: IT’S (CHANGE IN) THE (FUTURE) ECONOMY, STUPID. American Journal of Political Science. 2015 Feb;59(2):457–74.

[27] Young L, Soroka SN. Affective News: The Automated Coding of Sentiment in Political Texts. Political Communication. 2012 Apr;29(2):205–31.

[28] Rice DR, Zorn C. Corpus-based dictionaries for sentiment analysis of specialized vocabularies. PSRM. 2019 Apr 2;1–16.

[29] Nicholls T, Culpepper PD. Computational Identification of Media Frames: Strengths, Weaknesses, and Opportunities. Political Communication. 2020 Sep 18;1–23.

[30] Blitzer J, Dredze M, Pereira F. Biographies, Bollywood, Boom-boxes and Blenders: Domain Adaptation for Sentiment Classification. In: Proceedings of the 45th annual meeting of the Association of Computational Linguistics. 2007. p. 440–7.

[31] Lin B, Zampetti F, Bavota G, Di Penta M, Lanza M, Oliveto R. Sentiment analysis for software engineering: how far can we go? In: Proceedings of the 40th International Conference on Software Engineering [Internet]. Gothenburg Sweden: ACM; 2018 [cited 2024 Feb 27]. p. 94–104. Available from: https://dl.acm.org/doi/10.1145/3180155.3180195

[32] Lehrt D, Ohm P. Playing with the Data: What Legal Scholars Should Learn About Machine Learning. UC Davis Law Review. 2017;51(2):653–718.

[33] Hosking T, Blunsom P, Bartolo M. Human Feedback is not Gold Standard [Internet]. arXiv; 2024 [cited 2024 Feb 27]. Available from: http://arxiv.org/abs/2309.16349

[34] French RM. Catastrophic forgetting in connectionist networks. Trends in Cognitive Sciences. 1999;3(4). doi: 10.1016/s1364-6613(99)01294-2 10322466

[35] Zhai Y, Tong S, Li X, Cai M, Qu Q, Lee YJ, et al. Investigating the Catastrophic Forgetting in Multimodal Large Language Models.

[36] Kapoor S, Narayanan A. (Ir)Reproducible Machine Learning: A Case Study. Princeton, NJ: Princeton University; 2021 p. 7.

[37] Birhane A, Prabhu VU, Kahembwe E. Multimodal datasets: misogyny, pornography, and malignant stereotypes [Internet]. arXiv; 2021 [cited 2022 Dec 28]. Available from: http://arxiv.org/abs/2110.01963

[38] Gallegos IO, Rossi RA, Barrow J, Tanjim MM, Kim S, Dernoncourt F, et al. Bias and Fairness in Large Language Models: A Survey [Internet]. arXiv; 2024 [cited 2024 Aug 29]. Available from: http://arxiv.org/abs/2309.00770

[39] Bai X, Wang A, Sucholutsky I, Griffiths TL. Measuring Implicit Bias in Explicitly Unbiased Large Language Models [Internet]. arXiv; 2024 [cited 2024 Aug 29]. Available from: http://arxiv.org/abs/2402.04105

[40] Weidinger L, Mellor J, Rauh M, Griffin C, Uesato J, Huang PS, et al. Ethical and social risks of harm from Language Models [Internet]. arXiv; 2021 [cited 2024 Aug 29]. Available from: http://arxiv.org/abs/2112.04359

[41] González-Bailón S, Paltoglou G. Signals of Public Opinion in Online Communication: A Comparison of Methods and Data Sources. The ANNALS of the American Academy of Political and Social Science. 2015 May;659(1):95–107.

[42] Munikar M, Shakya S, Shrestha A. Fine-grained Sentiment Classification using BERT. In: 2019 Artificial Intelligence for Transforming Business and Society (AITB) [Internet]. Kathmandu, Nepal: IEEE; 2019 [cited 2024 Aug 30]. p. 1–5. Available from: https://ieeexplore.ieee.org/document/8947435/

[43] Cheang B, Wei B, Kogan D, Qiu H, Ahmed M. Language Representation Models for Fine-Grained Sentiment Classification [Internet]. arXiv; 2020 [cited 2024 Aug 30]. Available from: http://arxiv.org/abs/2005.13619

[44] Maleszka B. How to Explain Sentiment Polarity–A Survey of Explainable Sentiment Analysis Approaches. Cybernetics and Systems. 0(0):1–17.

[45] Bender EM, Gebru T, McMillan-Major A, Shmitchell S. On the Dangers of Stochastic Parrots: Can Language Models Be Too Big?. In: Proceedings of the 2021 ACM Conference on Fairness, Accountability, and Transparency [Internet]. Virtual Event Canada: ACM; 2021 [cited 2022 Dec 27]. p. 610–23. Available from: https://dl.acm.org/doi/10.1145/3442188.3445922

[46] Boukes M, van de Velde B, Araujo T, Vliegenthart R. What’s the Tone? Easy Doesn’t Do It: Analyzing Performance and Agreement Between Off-the-Shelf Sentiment Analysis Tools. Communication Methods and Measures. 2020 Apr 2;14(2):83–104.

[47] hong Chan C, Bajjalieh J, Auvil L, Wessler H, Althaus S, Welbers K, et al. Four best practices for measuring news sentiment using ‘off-the-shelf’ dictionaries: a large-scale p-hacking experiment. 1. 2021 Apr 13;3(1):1–27.

[48] Reagan AJ, Tivnan B, Williams JR, Danforth CM, Dodds PS. Benchmarking sentiment analysis methods for large-scale texts: A case for using continuum-scored words and word shift graphs. arXiv:151200531 [cs] [Internet]. 2015 Dec 1 [cited 2019 Feb 4]; Available from: http://arxiv.org/abs/1512.00531

[49] Taboada M, Brooke J, Tofiloski M, Voll K, Stede M. Lexicon-Based Methods for Sentiment Analysis. Computational Linguistics. 2011 Apr 5;37(2):267–307.

[50] Hu M, Liu B. Mining and Summarizing Customer Reviews. In: Proceedings of the tenth ACM SIGKDD International Conference on Knowledge Discovery and Data Mining. Seattle, WA; 2004. p. 168–77.

[51] Wilson T, Wiebe J, Hoffmann P. Recognizing Contextual Polarity in Phrase-Level Sentiment Analysis. In: Proceedings of Human Language Technology Conference and Conference on Empirical Methods in Natural Language Processing [Internet]. Vancouver, British Columbia, Canada: Association for Computational Linguistics; 2005 [cited 2020 Apr 3]. p. 347–54. Available from: https://www.aclweb.org/anthology/H05-1044

[52] Dodds PS, Harris KD, Kloumann IM, Bliss CA, Danforth CM. Temporal Patterns of Happiness and Information in a Global Social Network: Hedonometrics and Twitter. PLoS One [Internet]. 2011 Dec 7 [cited 2020 Apr 3];6(12). Available from: https://www.ncbi.nlm.nih.gov/pmc/articles/PMC3233600/10.1371/journal.pone.0026752 PMC3233600 22163266

[53] Mohammad SM, Yang TW. Tracking sentiment in mail: how genders differ on emotional axes. In: Proceedings of the 2nd workshop on computational approaches to subjectivity and sentiment analysis. 2011. p. 70–9.

[54] Baccianella S, Esuli A, Sebastiani F. SENTIWORDNET 3.0: An Enhanced Lexical Resource for Sentiment Analysis and Opinion Mining. In: Lrec vol 10. 2010. p. 2200–4.

[55] Provalis. Sentiment Analysis with WordStat [Internet]. Available from: https://provalisresearch.com/products/content-analysis-software/wordstat-dictionary/sentiment-dictionaries/

[56] Stone PJ, Hunt EB. A Computer Approach to Content Analysis: Studies Using the General Inquirer System. In: Proceedings of the May 21–23, 1963, Spring Joint Computer Conference [Internet]. New York, NY, USA: ACM; 1963 [cited 2019 Mar 6]. p. 241–56. (AFIPS ‘63 (Spring)). Available from: http://doi.acm.org/10.1145/1461551.1461583

[57] Martindale C. Romantic progression: The psychology of literary history. Washington, DC: Hemisphere; 1975.

[58] Soroka SN, McAdams S. News, Politics, and Negativity. Political Communication. 2015 Jan 2;32(1):1–22.

[59] Pang B, Lee L. A sentimental education: sentiment analysis using subjectivity summarization based on minimum cuts. In: Proceedings of the 42nd Annual Meeting on Association for Computational Linguistics—ACL ‘04 [Internet]. Barcelona, Spain: Association for Computational Linguistics; 2004 [cited 2020 Nov 23]. p. 271-es. Available from: http://portal.acm.org/citation.cfm?doid=1218955.1218990

[60] Maas AL, Daly RE, Pham PT, Huang D, Ng AY, Potts C. Learning word vectors for sentiment analysis. In: Proceedings of the 49th Annual Meeting of the Association for Computational Linguistics: Human Language Technologies—Volume 1. Association for Computational Linguistics; 2011. p. 142–50.

[61] Hendrycks D, Liu X, Wallace E, Dziedzic A, Krishnan R, Song D. Pretrained Transformers Improve Out-of-Distribution Robustness [Internet]. 2020 Apr [cited 2021 Jun 23]. Report No.: Arxiv 2004.06100. Available from: http://arxiv.org/abs/2004.06100

[62] Rhodes JH, Vayo AB. The Historical Presidency: Fear and Loathing in Presidential Candidate Rhetoric, 1952–2016. Presidential Studies Quarterly. 2019 Dec;49(4):909–31.

[63] Ribeiro FN, Araújo M, Gonçalves P, André Gonçalves M, Benevenuto F. SentiBench—a benchmark comparison of state-of-the-practice sentiment analysis methods. EPJ Data Science [Internet]. 2016 Dec [cited 2018 Aug 23];5(1). Available from: http://epjdatascience.springeropen.com/articles/10.1140/epjds/s13688-016-0085-1

[64] Barberá P, Boydstun AE, Linn S, McMahon R, Nagler J. Automated Text Classification of News Articles: A Practical Guide. Polit Anal. 2021 Jan;29(1):19–42.

[65] van der Veen AM. Automated text classification of news articles: Machine learning versus dictionaries. Williamsburg, VA: William & Mary; 2022.

[66] Lin B, Zampetti F, Oliveto R, Di Penta M, Lanza M, Bavota G. Two Datasets for Sentiment Analysis in Software Engineering. In: 2018 IEEE International Conference on Software Maintenance and Evolution (ICSME) [Internet]. Madrid: IEEE; 2018 [cited 2024 Feb 27]. p. 712–712. Available from: https://ieeexplore.ieee.org/document/8530087/

[67] Zhang T, Irsan IC, Thung F, Lo D. Revisiting Sentiment Analysis for Software Engineering in the Era of Large Language Models [Internet]. arXiv; 2023 [cited 2024 Feb 27]. Available from: http://arxiv.org/abs/2310.11113

[68] Touvron H, Martin L, Stone K, Albert P, Almahairi A, Babaei Y, et al. Llama 2: Open Foundation and Fine-Tuned Chat Models [Internet]. arXiv; 2023 [cited 2024 Mar 1]. Available from: http://arxiv.org/abs/2307.09288

[69] Chiang WL, Li Z, Lin Z, Sheng Y, Wu Z, Zhang H, et al. Vicuna: An Open-Source Chatbot Impressing GPT-4 with 90%* ChatGPT Quality [Internet]. 2023. Available from: https://lmsys.org/blog/2023-03-30-vicuna/

[70] Xu C, Sun Q, Zheng K, Geng X, Zhao P, Feng J, et al. WizardLM: Empowering Large Language Models to Follow Complex Instructions [Internet]. arXiv; 2023 [cited 2024 Mar 1]. Available from: http://arxiv.org/abs/2304.12244

[71] Jaspal R, Cinnirella M. Media representations of British Muslims and hybridised threats to identity. Cont Islam. 2010 Oct;4(3):289–310.

[72] Moore K, Mason P, Lewis J. Images of Islam in the UK: The Representation of British Muslims in the National Print News Media 2000–2008. Cardiff, UK: Cardiff School oof Journalism, Media and Cultural Studies; 2008 Jul p. 41.

[73] Morey P, Yaqin A. Framing Muslims: stereotyping and representation after 9/11. Cambridge, Mass: Harvard University Press; 2011. 246 p.

[74] Poole E. Reporting Islam [Internet]. I.B. Tauris; 2002 [cited 2019 Oct 25]. Available from: https://www.bloomsbury.com/us/reporting-islam-9781860646874/

[75] Richardson JE. ‘Get shot of the lot of them’: election reporting of Muslims in British newspapers. Patterns of Prejudice. 2009 Jul;43(3–4):355–77.

[76] Boomgaarden HG, Vliegenthart R. How news content influences anti-immigration attitudes: Germany, 1993–2005. European Journal of Political Research. 2009 Jun;48(4):516–42.

[77] Lajevardi N. Outsiders at Home: The Politics of American Islamophobia. Cambridge: Cambridge University Press; 2020.

[78] Saleem M, Prot S, Anderson CA, Lemieux AF. Exposure to Muslims in Media and Support for Public Policies Harming Muslims. Communication Research. 2017 Aug;44(6):841–69.

[79] Bail CA. The Fringe Effect: Civil Society Organizations and the Evolution of Media Discourse about Islam since the September 11th Attacks. Am Sociol Rev. 2012 Dec;77(6):855–79.

[80] Trevino M, Kanso AM, Nelson RA. Islam through editorial lenses: How American elite newspapers portrayed Muslims before and after September 11, 2001. journal of arab & muslim media research. 2010 Nov 1;3(1):3–17.

[81] Bleich E, Stonebraker H, Nisar H, Abdelhamid R. Media Portrayals of Minorities: Muslims in British Newspaper Headlines, 2001–2012. Journal of Ethnic and Migration Studies. 2015 May 12;41(6):942–62.

[82] Nacos BL, Torres-Reyna O. Framing Muslim-Americans before and after 9/11. In: Norris P, Kern M, Just M, editors. Framing Terrorism: The News Media, the Government and the Public [Internet]. New York, NY: Routledge; 2003. p. 133–58. Available from: https://books.google.com/books?id=EYzevgEACAAJ

[83] Connell I. Mistaken Identities: Tabloid and Broadsheet News Discourse. Javnost—The Public. 1998 Jan;5(3):11–31.

[84] Baker P. Representations of Islam in British broadsheet and tabloid newspapers 1999–2005. Journal of Language and Politics. 2010 Jul 23;9(2):310–38.

[85] Bleich E, van der Veen AM. Media portrayals of Muslims: a comparative sentiment analysis of American newspapers, 1996–2015. Politics, Groups, and Identities. 2018 Nov 8;1–20.

[86] Lefkowitz J. “Tabloidization” or Dual-Convergence: Quoted speech in tabloid and “quality” British newspapers 1970–2010. Journalism Studies. 2018 Feb 17;19(3):353–75.

