# Supplementary material for: The advantages of lexicon-based sentiment analysis in an age of machine learning
Source: PLoS One. 2025 Jan 10;20(1):e0313092. doi: 10.1371/journal.pone.0313092 (PMC11723603; doi:10.1371/journal.pone.0313092)
Supplement: S1 File — (DOCX) [file pone.0313092.s001.docx]

**The advantages of lexicon-based sentiment analysis
in an age of machine learning**

**Supporting information**

Contents

1. Newspaper selection

2. Article selection for representative corpora

3. Text pre-processing

4. Valence calculation

5. Additional validation data

6. Updated lexica starting Dec. 2021

References

1. Newspaper selection

For the British representative corpus, as well as for the Muslim corpus used in the latter part of the paper, we selected papers that have been in the top 20 of British papers by circulation for all or part of the period since 1996. Comparing circulation data is complicated in the UK, because many papers have separate or associated Sunday versions with different circulation figures. In addition, we aimed to get papers that were available for the entire 1996-2015 period, which was not always possible. Table S1 lists the papers for which we have full two-decade coverage; Table S2 those for which coverage starts later or ends earlier. The paper selection includes a wide representation of broadsheets as well as tabloids, along with a range of political leanings.

**Table S1. UK papers available for the entire period 1996-2015.**

|  | **Broadsheet** | **Tabloid** |
| --- | --- | --- |
| **Left-leaning** | *Guardian, Observer* | *Daily Record & Sunday Mail (Scotland),*  *Daily & Sunday Mirror, People* |
| **Centrist** | *Financial Times,*  *Independent* | *Evening Standard* |
| **Right-leaning** | *Times, Sunday Times* | *Daily Mail & Mail on Sunday* |

**Table S2. UK papers available for part of the period since 1996 only.**

| **Title** | **Broadsheet / Tabloid** | **Political leaning** | **Start date** | **End date** |
| --- | --- | --- | --- | --- |
| *Daily Star* | *T* | *R* | 1 Dec. 2000 |  |
| *Daily Star Sunday* | *T* | *R* | 15 Sep. 2002 |  |
| *The Daily Telegraph (London)* | *B* | *R* | 30 Oct 2000 |  |
| *The Sunday Telegraph (London)* | *B* | *R* | 5 Nov. 2000 |  |
| *The Express* | *T* | *R* | 13 Oct. 1999 (2006-2008 incomplete) |  |
| *i – Independent Print Ltd* | *B* | *C* | 28 Oct. 2010 |  |
| *News of the World* | *T* | *R* | 26 Jul. 1998  (2009 incomplete) | 3 Jul. 2011 |
| *The Sun (England)* | *T* | *R* | 31 Dec. 1999 |  |

For the United States representative corpus, we included both the largest-circulation national papers and leading regional papers, again ensuring inclusion of broadsheets as well as tabloids, as well as papers with different political leanings (Table S3). All papers except the *New York Post* are available online since 1996; the *Post* becomes available starting December 5, 1997.

**Table S3. US papers included in the representative corpus.**

| **National papers** | *New York Times, Washington Post, USA Today, Wall Street Journal* |
| --- | --- |
| **Regional papers** | *Atlanta Journal-Constitution, Boston Globe, Denver Post, Minneapolis Star-Tribune, Philadelphia Inquirer, Richmond Times Dispatch, San Jose Mercury News, Tampa Bay Times* |
| **Tabloids** | *New York Daily News, New York Post, Philadelphia Daily News* |

Finally, we presented data on smaller representative corpora for three additional English-language newspaper markets (Table S4). We selected 5-6 wide-circulation papers in each country. In the case of Canada, we selected papers from among the top 10 by circulation that are published in three different provinces; we added the *Vancouver Province* in order to include a tabloid title. For the other two countries, we simply selected the top 5 papers by circulation.

**Table S4. Papers included in representative corpora for 3 additional countries.**

| **Canada** | *The Gazette, Globe and Mail, Financial/National Post, Toronto Star, Vancouver Sun, Vancouver Province* |
| --- | --- |
| **Australia** | *Herald Sun* & *Sunday Herald Sun*, *Daily & Sunday Telegraph, Courier Mail & Sunday Mail, Sydney Morning Herald, West Australian* |
| **New Zealand** | *Dominion Post* (Aug. 2002-)*, New Zealand Herald, Otago Daily Times* (Aug. 2002-)*, The Press, Waikato Times* |

2. Article selection for representative corpora

Neither LexisNexis (now NexisUni) nor Factiva provide ways to randomly sample articles from a given source or within a particular time period. Moreover, Factiva requires a specific search term. To approximate a random sampling, we searched for words that do not have any valence themselves, under the assumption that the valence distribution across articles containing those words will mirror that of the distribution across all articles. Specifically, we selected from the labMT lexicon (see table S6 below) those words with a valence exactly at the midpoint of the lexicon’s scale that are also among the 4000 most common words in the English language. This produces a list of 18 words: because, per, standard, situation, carbon, assess, throw, liver, plain, supervisor, something, throat, whereas, boot, fourth, stir, price, and odds. LexisNexis does not permit a search for the word ‘because’, leaving us with 17 words. There is no reason to believe that articles in which these words appear are systematically biased towards positive or negative valences, nor that they are more or less distant from neutrality, and the validation results reported in the main text corroborates this.

Since these are all common words, a search over the full 20-year time range would be prohibitive. Instead, we searched for articles published on 3 randomly selected dates in each calendar year, ensuring that each half of the year contained at least one such date. (We began by selecting 2 random dates in each calendar year. For years in which those 2 dates fell within the same half of the year (January-June or July-December), we selected the third randomly from the other half. For all other years, the third date was selected randomly from the whole year.) A chronological listing of all selected dates appears in table S5 below. Each day of the week is represented, although Sundays appear only 3 times among the 60 dates. This means that British Sunday papers are somewhat under-represented in the sample. However, given shared editorial approaches between most Sunday papers and associated weekday papers, this is unlikely to affect any overall pattern.

**Table S5. Publication dates of articles in representative corpora.**

| 1996/2/27  1997/1/30  1998/6/5  1999/3/11  2000/3/29 | 1996/3/25  1997/8/18  1998/9/13  1999/3/17  2000/5/1 | 1996/12/9  1997/12/3  1998/10/27  1999/9/9  2000/10/16 |  | 2006/2/24  2007/2/24  2008/6/2  2009/5/18  2010/1/6 | 2006/8/24  2007/6/5  2008/7/2  2009/7/3  2010/5/25 | 2006/10/5  2007/10/12  2008/8/12  2009/10/27  2010/9/18 |
| --- | --- | --- | --- | --- | --- | --- |
| 2001/4/4  2002/3/21  2003/1/22  2004/5/14  2005/3/5 | 2001/10/3  2002/7/14  2003/2/15  2004/8/24  2005/5/9 | 2001/11/16  2002/8/21  2003/12/20  2004/12/18  2005/7/2 |  | 2011/5/14  2012/3/1  2013/3/1  2014/1/14  2015/3/26 | 2011/7/27  2012/4/28  2013/4/29  2014/3/13  2015/5/25 | 2011/8/7  2012/8/3  2013/12/16  2014/12/17  2015/7/28 |

3. Text pre-processing

All our newspaper articles were downloaded in text format from LexisNexis, ProQuest, or Factiva. From the output, we took the title and text body of each article and combined them to form the raw text. Next, we removed duplicates, comparing article titles and the beginnings of article texts to other titles/texts published by the same paper on the same day. We use Levenshtein string comparisons to weed out not only identical articles, but also those that might differ only by a word or two in the title or opening text. Since our newspaper article databases often contain several different editions for a given newspaper, de-duplication is essential.

Next, we performed a number of basic cleaning operations on each text. We converted sentence break punctuation such as question marks or exclamation points to periods; other forms of punctuation, such as commas, parentheses, etc. were removed. In addition, we surrounded special characters by spaces to set them off from words they might be attached to. Periods not marking sentence breaks (such as Mr., or U.S.) as well as possessives (the ‘s in “George’s book”) were removed and contractions were spelled out. Finally, we expanded some common abbreviations, such as those for months (Feb. becomes February).

**Translating texts into American English**

Most of the sentiment lexica we use were constructed from American English word lists. This means that some will overlook the same word spelled slightly differently: favour as opposed to favor; criticise as opposed to criticize, etc. In order to address this issue, we “translated” non-US newspaper articles into American English in several steps. First, we convert “–our” words to their American “–or” version, as appropriate. Next, we do the same for verb forms, from “–is” (-ise, -ising, -isation, etc.) to “–ize” and from “–lys” to “–lyz”. Finally, we take all the words in an extensive list of spelling variants available online at the Github repository for MultiLexScaled (formerly at <http://www.tysto.com/uk-us-spelling-list.html>). Any of these that are not covered by the first two steps are converted to American English as well.

4. Valence calculation

The MultiLexScaled sentiment analysis method is described in the paper. Here we provide some additional detail on the sentiment lexica we use, as well as on how we incorporate the effects of intensifiers such as negation words.

Each sentiment analysis dictionary has its own idiosyncrasies, shaped by the method used to generate the dictionary and the original application. This is true for domain-specific dictionaries as well as for general-purpose ones, although the latter can perform well across a range of applications [1,2]. Rather than rely on any single dictionary, we average sentiment scores produced by eight different, widely-used and individually validated dictionaries. These are: the lexicon produced by Liu and collaborators [3]; labMT, produced by Dodds and collaborators [4]; lexicoderSD, produced by Soroka and Young [5]; MPQA, produced by Wiebe and collaborators [6]; NRC, developed at the Canadian National Research Council [7]; SentiWordNet, based on WordNet [8]; SO-CAL, developed by Taboada and collaborators [9]; and WordStat, constructed by Provalis Research [10]. Table S6. offers more information about each of these.

**Table S6. Sentiment analysis lexica used.**

| **Name** | **Positive**  **terms** | **Negative**  **terms** | **Notes on lexicon construction** |
| --- | --- | --- | --- |
| HuLiu | 2003 (+1) | 4782 (-1) | Constructed at the University of Illinois in Chicago, based on WordNet [11]. Developed for social media; contains terms such as “f*ck”. |
| labMT | 2668  (range from 1 to 3.5) | 1063 (range from -1 to -3.5) | Mechanical Turk coders coded the ‘happiness level’ of the most frequent 5,000 words from four separate sources: Twitter, Google Books (English), music lyrics (1960 to 2007), and the *New York Times* (1987 to 2007). Full lexicon has 10,222 entries. We filter out words with low valence scores (absolute value < 1), as recommended by the lexicon’s creators. |
| LexicoderSD 2015 | 1608 (+1), of which 1040 stems | 2745 (-1), of which 1958 stems | All words from the General Inquirer (GI) [12], the Regressive Imagery Dictionary (RID) [13], and Roget’s Thesaurus with the same valence in all 3 dictionaries (or same in 2 and omitted from the third). Includes wildcards to accept any endings for a given stem. |
| MPQA | 2299 (range from 0.175 to 1) | 4150 (range from  -0.175 to -1) | Used words from GI, from [14], and from their own prior work [15]. We use only single-word entries (no phrases), and average valence for words with multiple entries. ‘strong’ polarity is given a value of 1, ‘weak’ polarity gets ½. |
| NRC | 2227 (+1) | 3235 (-1) | All words from Roget’s thesaurus that occur at least 120,000 times in Google’s n-gram corpus, coded using 5 different MT coders for each word. |
| SentiWordNet | 11116 (range from 0.1 to 1) | 13106 (range from -0.1 to -1) | Assigns valences to the synonym sets (synsets) in the online semantic dictionary WordNet. Starting from ‘paradigmatically’ positive or negative words, propagated valence across WordNet using the network structure implied by synsets sharing words. Full lexicon has 29,436 entries; we filter out words with low aggregate valence (absolute value < 0.1). For words with multiple valences (e.g. in multiple synsets), we average the values. |
| SO-CAL | 3706 (range from 0.5 to 5.0) | 6306 (range from -0.5 to -5.0) | “Sentiment Orientation CALculator”, manually constructed from all words in a 400-text corpus of Epinions reviews, movie reviews [16], and GI. |
| WordStat 2.0 | 4623 (+1), of which 325 stems | 9182 (-1), of which 565 stems | Constructed by Provalis (makers of WordStat), by combining word lists from GI, RID, and the Linguistic and Word Count dictionary (LIWC) [17] and searching WordStat’s internal dictionary for potential synonyms. Includes wildcards to specify any ending acceptable for a given stem. |

On any given sentiment analysis application, one of these dictionaries will perform best. However, it is impossible to know *ex ante* which dictionary that will be. Taking the average score, therefore, will produce a robust measure that is far less vulnerable to the particularities of a given corpus of texts than any single dictionary would be.

**Intensifiers**

Benamara et al. [18] offer a broad survey of ways to extend a basic bag of words approach — to take into account the order in which words appear, in other words. The most common approach has been to take into account intensifiers, including negators, that directly precede sentiment words [18,19]. Doing so is relatively straightforward and makes intuitive sense. Accordingly, we adopt the method proposed by Taboada et al. [9], adjusting a word’s value in the lexicon based on any intensifiers that directly precede the word in the text. Specifically, we use a list of 216 different intensifiers and apply their associated modifying factor to the subsequent valence word. Some of these intensify the strength of a word, some weaken it, and some change the polarity. We handle multiple consecutive intensifiers (including negation) simply by combining their individual intensification effects.

The default multiplier for a valence word is 1. Intensifier values are added to this default, and the result is multiplied by the valence. For example, “slightly” has a multiplier of ‑0.5, which means that a subsequent word’s valence is multiplied by (1 + -0.5) = 0.5. We handle negation in a parallel fashion by identifying words that shift polarity, such as not, no, nor, nothing, never, and nowhere, and add them to the list of polarity-shifting words in Taboada et al., such as “hardly.” To get a polarity shift, we need a multiplier below -1. We assign our negation and polarity-shifting words a multiplier of -1.5, so that the valence is multiplied by (1 + -1.5) = -0.5.^[[1]](#footnote-1)^ The intensification multiplier is applied to the next valence word; it resets upon encountering a word that is not either another intensifier, a valence word, or a stopword we can skip over.

Table S7 compares our approach to the simpler alternatives of considering only negations (which simply reverse polarity) or not taking into account valence shifters at all. As the table makes clear, our approach offers an improvement over the alternatives, but the improvement is not great. Interestingly, compared to ignoring all modifiers, taking into account negations actually worsens overall performance, possibly because negation is never quite the same as inversion (“not good” is not the same as “bad”)

**Table S7. Validation test results on the imdb corpus: negators, intensifiers**

| **Adjustments to approach** | **% correct** | **Worst lexicon** | **% correct** | **Best lexicon** | **% correct** |
| --- | --- | --- | --- | --- | --- |
| - | 75.68 | labMT | 67.97 | SO-CAL | 78.13 |
| Negations only  (no other intensifiers) | 74.87 | labMT | 65.54 | SO-CAL | 77.61 |
| No intensif./negations | 75.03 | NRC | 67.20 | SO-CAL | 76.70 |

5. Additional validation data

In introducing the imdb dataset, Maas et al. reported results from a variety of machine learning approaches. These had the advantage of learning from half of the dataset (25,000 articles, including 12,500 of each polarity) before being tested on the other half. Results ranged from 67% to nearly 90% accuracy [20]. It is not surprising that a dedicated, corpus-specific machine learning algorithm would outperform ours; what is noteworthy is that our approach outperformed any such models at all, as it did. Moreover, in other published work using the same dataset, Khan et al. combine a lexicon-based approach (using SentiWordNet) with machine learning and still do less well than our approach presented here, attaining 75.22% accuracy [21].

In table S8, we report the method’s performance on three smaller datasets with more political texts (as opposed to movie reviews), comparing it against some other well-known approaches. Doing so is greatly facilitated by Ribeiro et al.’s work on SentiBench, which compares 24 “state-of-the-practice” (as of 2016) methods [22]. Due in part to burgeoning interest in social media sentiment, many benchmark datasets contain sentence-length text snippets with emoticons and other special punctuation, whereas our method was designed with longer, more formal texts in mind. In fact, other scholars have argued that it is effectively impossible for lexicon-based approaches to perform well on individual sentence-length snippets [23]. Nonetheless, these tests offer a useful indicator of the general strength of MultiLexScaled.

The first dataset consists of debate tweets (1249 negative and 730 positive) collected during the presidential debate between Barack Obama and John McCain in 2008, during the early days of Twitter. Many of these tweets contain little or no sentiment, making it difficult to assess whether the sentiment polarity (positive or negative) is accurately coded. The SentiBench approach does not penalize sentiment classifiers for not classifying a text; to compare against our method directly, we assume that each method would classify half of the unclassified tweets as positive and half as negative. Our method classified 70.64% of the tweets correctly, outperforming all 24 of the other sentiment classifiers tested in SentiBench, both lexicon-based as well as machine-learning approaches, including some specially designed for Twitter.

The second dataset is more directly comparable to our intended application of general, non-colloquial texts. It is a collection of news-related discussion posts “about various serious topics” on the BBC’s online Forum [24]. The dataset contains 653 negative posts and 99 positive ones. On these texts, our method correctly classified 73.67%. This outperforms 22 of the 24 sentiment classifiers tested by Ribeiro et al. Finally, we test our method on 2204 positive and 2272 negative sentence-length snippets from *New York Times* op-eds [25]. Here, again, our method outperforms all those tested by Ribeiro et al. It is worth noting that the best-performing lexicon is different in each case, underscoring the value of averaging across a number of lexica to maximize cross-application performance.

| **Labeled corpus** | **% correct** | **Worst**  **lexicon** | **% correct** | **Best lexicon** | **% correct** | **Ranking  (vs. 24 other methods)** |
| --- | --- | --- | --- | --- | --- | --- |
| Debate tweets | 70.64 | NRC | 62.10 | SO-CAL | 72.12 | 1 of 25 |
| BBC comments | 73.67 | SentiWordNet | 60.64 | HuLiu | 77.53 | 3 of 25 |
| NYT comments | 70.80 | SentiWordNet | 60.09 | Wordstat | 73.02 | 1 of 25 |

Table S8. Additional validation tests (accuracy compared against benchmarks in [22]).

6. Updated lexica starting December 2021

All the lexica used in MultiLexScaled are publicly available; URLs and the necessary preprocessing code are supplied in the replication materials. However, two of the lexica were used in earlier formats prior to December 2021. First, the SO-CAL lexicon features singular versions of nouns and the infinitive form of verbs. The initial version of MultiLexScaled added only some plurals and verb conjugations; the current version does so more systematically, using the python module *pattern* [26]. In addition, Provalis has released a new version of its WordStat sentiment lexicon, with the original version no longer readily available. The current version accordingly uses this new version of the lexicon.

The result of these two changes for sentiment classification accuracy is negligible; sentiment levels become somewhat more dispersed, since both the SO-CAL and WordStat dictionaries now capture more words. For comparison, using the original versions of these two lexica lowers accuracy on the imdb movie review dataset from 75.68 to 75.40. Meanwhile, the mean valence for the British Muslim newspaper corpus using the original version was -0.95; with the current version of MultiLexScaled it is -1.07.

References

1. Blitzer J, Dredze M, Pereira F. Biographies, Bollywood, Boom-boxes and Blenders: Domain Adaptation for Sentiment Classiﬁcation. In: Proceedings of the 45th annual meeting of the Association of Computational Linguistics. 2007. p. 440–7.

2. González-Bailón S, Paltoglou G. Signals of Public Opinion in Online Communication: A Comparison of Methods and Data Sources. Ann Am Acad Pol Soc Sci. 2015 May;659(1):95–107.

3. Hu M, Liu B. Mining and Summarizing Customer Reviews. In: Proceedings of the tenth ACM SIGKDD International Conference on Knowledge Discovery and Data Mining. Seattle, WA; 2004. p. 168–77.

4. Dodds PS, Harris KD, Kloumann IM, Bliss CA, Danforth CM. Temporal Patterns of Happiness and Information in a Global Social Network: Hedonometrics and Twitter. PLoS ONE [Internet]. 2011 Dec 7 [cited 2020 Apr 3];6(12). Available from: https://www.ncbi.nlm.nih.gov/pmc/articles/PMC3233600/

5. Young L, Soroka SN. Affective News: The Automated Coding of Sentiment in Political Texts. Polit Commun. 2012 Apr;29(2):205–31.

6. Wilson T, Wiebe J, Hoffmann P. Recognizing Contextual Polarity in Phrase-Level Sentiment Analysis. In: Proceedings of Human Language Technology Conference and Conference on Empirical Methods in Natural Language Processing [Internet]. Vancouver, British Columbia, Canada: Association for Computational Linguistics; 2005 [cited 2020 Apr 3]. p. 347–54. Available from: https://www.aclweb.org/anthology/H05-1044

7. Mohammad SM, Yang TW. Tracking sentiment in mail: how genders differ on emotional axes. In: Proceedings of the 2nd workshop on computational approaches to subjectivity and sentiment analysis. 2011. p. 70–9.

8. Baccianella S, Esuli A, Sebastiani F. SENTIWORDNET 3.0: An Enhanced Lexical Resource for Sentiment Analysis and Opinion Mining. In: Lrec vol 10. 2010. p. 2200–4.

9. Taboada M, Brooke J, Tofiloski M, Voll K, Stede M. Lexicon-Based Methods for Sentiment Analysis. Comput Linguist. 2011 Apr 5;37(2):267–307.

10. Provalis. Sentiment Analysis with WordStat [Internet]. Available from: https://provalisresearch.com/products/content-analysis-software/wordstat-dictionary/sentiment-dictionaries/

11. Miller GA. WordNet: a lexical database for English. Commun ACM. 1995 Nov 1;38(11):39–41.

12. Stone PJ, Hunt EB. A Computer Approach to Content Analysis: Studies Using the General Inquirer System. In: Proceedings of the May 21-23, 1963, Spring Joint Computer Conference [Internet]. New York, NY, USA: ACM; 1963 [cited 2019 Mar 6]. p. 241–56. (AFIPS ’63 (Spring)). Available from: http://doi.acm.org/10.1145/1461551.1461583

13. Martindale C. Romantic progression: The psychology of literary history. Washington, DC: Hemisphere; 1975.

14. Hatzivassiloglou V, McKeown KR. Predicting the Semantic Orientation of Adjectives. In 1997. p. 174–81.

15. Riloff E, Wiebe J. Learning extraction patterns for subjective expressions. In: Proceedings of the 2003 conference on Empirical methods in natural language processing - [Internet]. Not Known: Association for Computational Linguistics; 2003 [cited 2020 Aug 15]. p. 105–12. Available from: http://portal.acm.org/citation.cfm?doid=1119355.1119369

16. Pang B, Lee L, Vaithyanathan S. Thumbs up? Sentiment Classification using Machine Learning Techniques. In: EMNLP-2002 [Internet]. 2002 [cited 2020 Aug 15]. Available from: http://arxiv.org/abs/cs/0205070

17. Tausczik YR, Pennebaker JW. The Psychological Meaning of Words: LIWC and Computerized Text Analysis Methods. J Lang Soc Psychol. 2010 Mar;29(1):24–54.

18. Benamara F, Cesarano C, Picariello A, Reforgiato D, Subrahmanian V. Sentiment Analysis: Adjectives and Adverbs are better than Adjectives Alone. In: ICWSM. Boulder, CO; 2006. p. 7.

19. Kennedy A, Inkpen D. Sentiment classification of moview reviews using contextual valence shifters. Comput Intell. 2006;22(2):110–25.

20. Maas AL, Daly RE, Pham PT, Huang D, Ng AY, Potts C. Learning word vectors for sentiment analysis. In: Proceedings of the 49th Annual Meeting of the Association for Computational Linguistics: Human Language Technologies - Volume 1. Association for Computational Linguistics; 2011. p. 142–50.

21. Khan FH, Qamar U, Bashir S. SentiMI: Introducing point-wise mutual information with SentiWordNet to improve sentiment polarity detection. Appl Soft Comput. 2016 Feb;39:140–53.

22. Ribeiro FN, Araújo M, Gonçalves P, André Gonçalves M, Benevenuto F. SentiBench - a benchmark comparison of state-of-the-practice sentiment analysis methods. EPJ Data Sci [Internet]. 2016 Dec [cited 2018 Aug 23];5(1). Available from: http://epjdatascience.springeropen.com/articles/10.1140/epjds/s13688-016-0085-1

23. Reagan AJ, Tivnan B, Williams JR, Danforth CM, Dodds PS. Benchmarking sentiment analysis methods for large-scale texts: A case for using continuum-scored words and word shift graphs. ArXiv151200531 Cs [Internet]. 2015 Dec 1 [cited 2019 Feb 4]; Available from: http://arxiv.org/abs/1512.00531

24. Thelwall M, Buckley K, Paltoglou G. Sentiment strength detection for the social web. J Am Soc Inf Sci Technol. 2012 Jan;63(1):163–73.

25. Hutto CJ, Gilbert E. VADER: A Parsimonious Rule-based Model for Sentiment Analysis of Social Media Text. In: Proceedings of ICWSM-14. Ann Arbor, MI; 2014. p. 10.

26. De Smedt T, Daelemans W. Pattern for Python. J Mach Learn Res. 2012;13(66):2063–7.

1. The intuition here is that “not good” is generally not as bad as “bad” (nor is “not bad” the same as “good”), so simply reversing polarity is not the appropriate adjustment. [↑](#footnote-ref-1)
